# Winter plankton dynamics in a boreal lake: community structure, vertical distribution and reproduction under ice

**DOI:** 10.1093/plankt/fbaf035

**Published:** 2025-08-17

**Authors:** Jean-Simon Boulianne, E Henriikka Kivilä, Beatrix E Beisner, Milla Rautio

**Affiliations:** Département des Sciences Fondamentales, Université du Québec à Chicoutimi, 555 Boulevard de l’Université, Chicoutimi, Québec G7H 2B1, Canada; Groupe de Recherche Interuniversitaire en Limnologie, Université de Montréal, C.P. 6128 Succursale Centre-ville, Montréal, Québec H3C 3J7, Canada; Centre d'études nordiques (CEN), Quebec City, QC G1V 0A6, Canada; Département des Sciences Fondamentales, Université du Québec à Chicoutimi, 555 Boulevard de l’Université, Chicoutimi, Québec G7H 2B1, Canada; Groupe de Recherche Interuniversitaire en Limnologie, Université de Montréal, C.P. 6128 Succursale Centre-ville, Montréal, Québec H3C 3J7, Canada; Centre d'études nordiques (CEN), Quebec City, QC G1V 0A6, Canada; Groupe de Recherche Interuniversitaire en Limnologie, Université de Montréal, C.P. 6128 Succursale Centre-ville, Montréal, Québec H3C 3J7, Canada; Département des Sciences Biologiques, Université du Québec à Montréal, 141 Avenue du Président-Kennedy, Montréal, Québec, H2X 1Y4, Canada; Département des Sciences Fondamentales, Université du Québec à Chicoutimi, 555 Boulevard de l’Université, Chicoutimi, Québec G7H 2B1, Canada; Groupe de Recherche Interuniversitaire en Limnologie, Université de Montréal, C.P. 6128 Succursale Centre-ville, Montréal, Québec H3C 3J7, Canada; Centre d'études nordiques (CEN), Quebec City, QC G1V 0A6, Canada

**Keywords:** winter limnology, heterotrophy, zooplankton, under-ice vertical distribution, lake ice, zooplankton reproduction

## Abstract

Lakes are understudied during ice-covered periods; yet physical, hydrodynamical and biological processes continue under the ice. Ice cover reorganizes lake ecosystems by altering thermal stratification and solar radiation, creating conditions that support different organisms, food webs, and potential trophic cascades. We investigated seasonal shifts in biomass and vertical distribution of heterotrophic and autotrophic micro-organisms (heterotrophic bacteria, picoautotrophs, colorless and pigmented nanoflagellates) and zooplankton across winter in relation to limnological characteristics and hydrodynamics. Boreal Lake Simoncouche was sampled at five depths on six dates between autumn overturn (November 2020) and spring overturn (April 2021) for temperature, dissolved oxygen, chlorophyll-*a*, and plankton. Low chlorophyll-*a* and high heterotroph-to-autotroph biomass ratios indicated dominance of the heterotrophic energy pathway under ice. Heterotrophic micro-organisms also dominated during well-lit overturns, emphasizing the role of the microbial loop in all seasons. Zooplankton richness remained high under ice (18–22 taxa), with most species favoring deeper, warmer layers. Several species of rotifer and cladoceran sustained egg production through winter, ceasing only briefly in February. These findings highlight strong vertical and seasonal heterogeneity in winter-active plankton communities, shaped by stratification, light, and resources, and support the view that winter is biologically dynamic, with consequences for the subsequent open-water season.

## INTRODUCTION

Plankton are sensitive to fluctuations in temperature, light and oxygen, and winter is a key driver of major shifts in these environmental variables (Hampton *et al.,* 2017; Cavaliere *et al.,* 2021; Jansen *et al.,* 2021). Globally, ~86% of all lakes are covered by ice and snow for a period of at least one month each year (Korver *et al.,* 2024). Around 62% of earth’s 1.4 million lakes (>10 ha) are located in Canada, and are seasonally subjected to freezing temperatures (Messager *et al.,* 2016). The water column is affected to a great extent by the presence of an ice layer, which forms a physical barrier and seals the lake from its catchment and from the atmosphere (Cavaliere *et al.,* 2021). The ice cover prevents gas exchanges and reduces light penetration in the water column and modulates physical, chemical, and biological parameters. Phases of black—*i.e.* transparent—and white—*i.e*. opaque—ice formation, build-up and melting of snow cover, and ice melting, along with consequent changes in light and thermal regimes, divide the season into distinct periods. The extent and severity of these phases of the ice-covered season have the potential to influence ecological dynamics during the following open-water season (Hébert *et al.,* 2021; Jansen *et al.,* 2021). Furthermore, climate change is disproportionately affecting higher latitude lakes with the duration of ice cover decreasing at a rate of 17 ± 6.5 days (SD) per century, including more intermittent ice cover and rain on ice (Magnuson *et al.,* 2000; Sharma *et al.,* 2019; Sharma *et al.,* 2021). Such changes are expected to profoundly impact lake ecology during winter and into the following open water period (Hébert *et al.,* 2021; Hrycik and Stockwell, 2021; Socha *et al.,* 2023).

Aquatic winter ecology remains poorly understood, and the community dynamics of zooplankton under ice are still largely unknown (Sutton *et al.,* 2021). The Plankton Ecology Group (PEG) model originally assumed that plankton communities in the spring start from close to zero density and viewed winter as a biologically inactive season (Sommer *et al.,* 1986). The model has since been updated but still does not account for the heterogeneity and progression of winter (Sommer *et al.,* 2012; Cavaliere *et al.,* 2021). Baseline data are needed to construct adequate models of the seasonality of lakes and to better predict and anticipate the effects of climate change on plankton communities of lakes that freeze.

The objectives of this study were to characterize the vertical structure of Lake Simoncouche during the ice-covered period and adjacent autumn and spring shoulder seasons, and to examine seasonal patterns in energy pathways, zooplankton community structure, and zooplankton reproduction. We first hypothesized (H1) that seasonality and depth would affect photo-autotrophic biomass: specifically given declining solar radiation, we predicted a decrease in photo-autotrophic biomass with progressing autumn into winter and with water column depth. Second, we hypothesized (H2) shifts in zooplankton biomass in the winter owing to altered phytoplankton biomass. We thus predicted overall winter declines in biomass of all zooplankton groups (rotifers, copepods and cladocerans), linked to reduced phytoplankton resources and lower water column heat content, which limit zooplankton survival and production (Brown *et al.*, 2004; Sommer *et al.,* 2012). Third, we hypothesized (H3) that vertical variability in zooplankton biomass, including relative biomass of different taxa, would be shaped by a combination of thermal stratification and food availability. Zooplankton should be attracted to warmer water at deeper depths in mid-winter, but remain closer to the surface in melting season upon the return of autotrophic production with increasing solar radiation through metamorphosing and melting ice (Devkota *et al*., 2024; Jansen *et al.,* 2021). Fourth, we hypothesized (H4) a strong effect of the end of winter on copepod nauplii and cladoceran juvenile abundances. We expected greater abundances with peaks in production near the end of winter, but before the spring algal bloom, reflecting under-ice reproduction and active overwintering strategies of some zooplankton taxa (Grosbois and Rautio, 2017; Mariash *et al*., 2016). Finally, we hypothesized (H5) that rotifers reproduce under ice, consistent with their resilience in winter environments (Virro *et al*., 2009; Kalinowska and Karpowicz, 2020).

## METHOD

This study was conducted during winter 2020–2021 at boreal Lake Simoncouche (48° 13′ 57″ N, 71° 14′ 57″ W). The lake is small (0.8 km^2^) dimictic and mesotrophic (mean total dissolved phosphorous and nitrogen concentrations during the sampling season were, respectively, 7.2 μg L^−1^ and 0.36 mg L^−1^; Kivilä *et al.,* 2023), characterized by a short mean residence time of 50 days, and has a mean depth of 2.2 m and a maximum depth of 8 m. The lake is annually ice-covered for five to six months, usually between mid-November and early May. Maximum ice thickness rarely exceeds 1 m and was 43 cm during the winter of 2020–2021. The ice cover comprises mostly white ice (up to 75%) and was overlain by a snow layer that ranged from 10 to 20 cm during the sampling period (Kivilä *et al.,* 2023). Species of calanoid and cyclopoid copepods, cladocerans, and rotifers compose the zooplankton community and the most common crustacean species in the lake, *L. minutus,* reproduces under ice (Grosbois *et al.,* 2017; Grosbois and Rautio, 2017; Schneider *et al.,* 2017).

We sampled four times during the ice-covered season, from 14 December 2020 to 12 April 2021, at five fixed depths to test H1 to H3. The lake was also sampled during the autumn overturn period, 21 days before ice-on (13 November) and during the spring overturn period, 7 days after ice-off (19 April). Sampling dates when the lake was frozen were chosen to overlap key winter time periods, *sensu*  Jansen *et al.* (2021): early winter (14 December), mid-winter (23 February), late winter (23 March) and melting season (12 April). Five depths across the whole water column were sampled at the deepest point of the lake (*Z*_max_ = 8 m) at 0 m, (immediately below the ice), 1.5 m, 3 m, 4.5 m, and 7 m, as measured from the top of the ice and excluding snow. All samples were taken at these same depths, except during the overturn periods when integrated samples of the whole water column were taken given vertical homogeneity at these times in temperature, oxygen and conductivity.

Physical water column properties (temperature, dissolved oxygen saturation, specific conductivity) were profiled with a Ruskin RBR Concerto multi-channel logger (RBR Ltd, Canada) on each sampling date. Two RBR Maestro loggers (1.5 m and 6 m depths) were additionally set to record temperature, photosynthetically active radiation (PAR), O_2_ saturation and specific conductivity, at 30-minute intervals throughout winter 2020–2021 (Supplementary Material S1: Fig. S1). The optical sensors (O_2_ and PAR) were equipped with wipers that cleaned them every 30 min.

Sample water was collected around noon with a 2 L Limnos sampler (Limnos Ltd, Poland) and transferred into 4 L opaque Nalgene bottles (three replicates per depth) for chlorophyll-*a* (chl-*a*), nanoflagellates, bacterioplankton, and autotrophic picoplankton (Supplementary Material S2); all data relevant to testing H1-H3. Sample preparation in the laboratory was done on the same day as sampling. For chl-*a* analyses, 200–350 mL of lake water was filtered through a 25-mm GF/F filter (Whatman, UK; pore size 0.7 μm) and stored frozen (−80°C) until extracted in ethanol and analyzed with a Cary Eclipse spectrofluorometer (Agilent Technologies, USA) according to Nusch (1980). The extracts were scanned before and after acidification to subtract phaeopigments.

To test H1 regarding photo-autotroph activity, nanoflagellate (NF) samples were prepared immediately after arriving at the laboratory; a 50-mL aliquot was preserved with 5 mL filtered (< 0.2 um) glutaraldehyde solution (1% final concentration). Preserved samples were stored at 4°C and slides were prepared within 48 h. The NF slides were made from a volume of 25 mL of lake water and were stained with nucleic acid–staining fluorochrome 4′,6-diamidino-2-phenylindole (DAPI) prior to filtration through a 0.6-μm black polycarbonate membrane (Whatman, UK) and permanent mounting on microscope slides with immersion oil, as in Rautio *et al.* (2011). Slides were stored at −20°C until enumeration of the microbial organisms at 100X magnification using an Axio Observer A1 (Zeiss, Germany) microscope with ultraviolet (UV) illumination and light filters to separate between heterotrophic (HNF) and pigmented NF (PNF). Nanoflagellates were enumerated in three different size categories (diameter 2–5, 5–10, and > 10 μm) to allow volume estimations and subsequent carbon content for biomass conversion, using a conversion factor of 0.22 pg C μm^−3^ (Børsheim *et al.,* 1990).

Zooplankton samples were concentrated from a volume of 20 L of lake water taken at each sampling depth with the Limnos sampler, allowing us to test hypotheses H2–H5. This volume ensured that the samples contained a minimum number of 400 rotifers and crustacean individuals, which were considered statistically reliable and representative in estimating community composition, diversity, and abundance. Samples were filtered using a 50-μm mesh net transferred to a 250-mL Nalgene bottle. A few drops of formaldehyde were added in the field to euthanize the organisms, and more (4% final concentration) in the laboratory. Two zooplankton sample replicates were collected at each depth. Zooplankton were counted after sedimentation for 24 hours in Utermöhl sedimentation chambers using an inverted microscope in white light illumination (Axio Observer, A1, Zeiss, Germany, 10–100X). Taxa identification was done at the highest possible taxonomic resolution using taxonomic keys from Edmondson (1959) and Czaika (1982), and an online image-based taxonomic key (Haney *et al.,* 2013). The number of organisms per taxa and per developmental stage was recorded, generating abundances (10^3^ ind. m^−3^) for each taxon by date and by depth, as a mean (± standard error) of the two replicates. Rotifers, cladocerans and copepods carrying eggs were also counted as percentage of population to test H4 and H5. Ten individuals were measured for each taxon and development stage across all samples using an optical camera (AxioCam ERC 5S, Zeiss, Germany) and microscope software (AxioVision, Zeiss, Germany). The mean of the ten length measures per taxa was used in the biomass (dry weight, DW) conversion to estimate the mean DW of each individual using taxa-specific length-DW regressions (Bottrell *et al.,* 1976; Lawrence *et al.,* 1978; McCauley, 1984; Culver *et al.,* 1985).

To evaluate the effects of time (4 levels, fixed) and depth (5 levels, fixed) on under-ice rotifer, calanoid, cyclopoid and cladoceran biomasses and heterotrophic:autotrophic nanoflagellate HNF:PNF mass ratio, we conducted univariate (*i.e.* one variable at a time) permutational analysis of variance (PER-ANOVA) using Euclidean distance matrices, following methods from Anderson *et al.* (2008). Zooplankton biomasses and the HNF:PNF mass ratio were Log(x + 1) transformed prior to analyses to account for zero values. These analyses directly addressed our hypotheses regarding seasonal (H2) and vertical variation (H3) in energy pathways and zooplankton productivity under the ice, particularly associated with the expected decline in autotrophic biomass and increase in heterotrophic dominance over winter (H1).

To assess zooplankton community structure associated with time and depth changes (H3), we used a permutational multivariate analysis of variance (PERMANOVA) (Anderson, 2014) with the same time × depth design. Taxa-specific biomass data were log-transformed and dispersion-weighted, and Bray–Curtis dissimilarities were used to construct the distance matrix. This analysis was designed to test our hypothesis that vertical and seasonal gradients, including thermal stratification and resource availability, shape the composition of winter-active zooplankton communities. Where significant effects were detected, post-hoc permutation t-tests were performed. We also tested for interactions between depth and time to evaluate whether community changes were synchronous across depths or differed in magnitude and timing.

To visualize changes in zooplankton assemblages across depths and time points, we performed a non-metric multidimensional scaling (nMDS) ordination based on Bray-Curtis dissimilarities. To further interpret group differences, SIMPER analysis was conducted to identify zooplankton taxa contributing most to dissimilarities among time periods and depths. These community-level approaches supported hypothesis testing related to adaptive overwintering strategies and taxon-specific responses to changing environmental conditions under ice (H4 and H5).

The software JMP (version 14.3) was used for all univariate tests while PRIMER + PERMANOVA (version 7.0.1) was used for the multivariate analyses, with a significance level of α = 0.05 for all analyses. Data visualizations were produced with R statistical software (R Core Team 2023) and ggplot2 package (Wickham, 2016), except the nMDS for which PRIMER was used.

## RESULTS

**Fig. 1:**
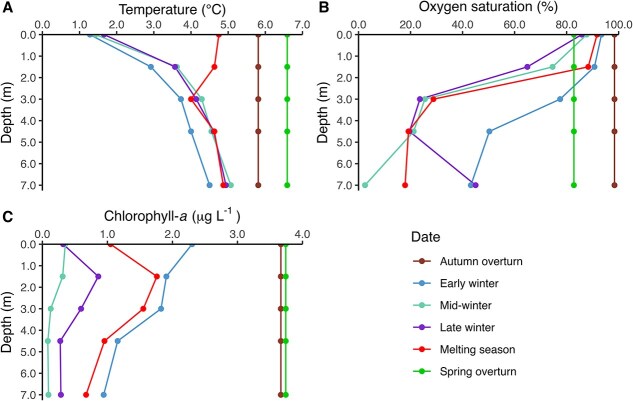
Water column profiles of temperature (**A**), oxygen saturation (**B**) and chlorophyll-*a* concentration (**C**) for the six sampling periods (autumn overturn, early, mid, and late winter, melting season, spring overturn) during winter 2020–2021 in Lake Simoncouche. Temperature (°C) and oxygen saturation (%) measurements were taken using a RBR Concerto multi sensor profiler, while chlorophyll-*a* concentrations (μg L^-1^) represent the means of three replicates determined by extraction from water samples at five sampling depths.

Lake Simoncouche was thermally stratified during winter 2020–2021, with temperatures colder at the surface and warmer at the bottom (Fig. 1A). Surface waters were coldest in early winter at 1.3°C and increased to 4.8°C in the melting season, while the deepest stratum remained stable throughout winter, with temperatures ranging between 4°C and 5°C. During the fall and spring overturns, water column temperature was homogeneous (5.8°C in autumn and 6.6°C in spring) as full thermal mixing occurred. Oxygen saturation was generally higher in the upper strata (Fig. 1B), gradually decreasing at the surface as winter progressed but never below 85%. The saturation in the bottom stratum declined to a minimum of 2.5% in mid-winter, increased to 45% by late winter, likely due to O_2_-rich intrusions from the littoral zone reaching the profundal, then declining again to 18% in the melting season. Dissolved O_2_ was homogeneous and 98% saturated during the autumn overturn, while levels were 83% in the spring overturn period. Chlorophyll-*a* concentrations were highest during the overturns at ~3.7 μg L^−1^ (Fig. 1C). During the ice-covered period, water column concentrations of chlorophyll-*a* peaked in early winter (2.3 μg L^−1^ at 0 m), decreasing at mid-winter (0.35 μg L^−1^ at 0 m) before increasing again toward late winter and the melting season, when PAR in the upper water column also increased (Supplementary Material S1: Fig. S1b). In early and mid-winter, the surface layer contained greater chlorophyll-*a* concentrations than other depths, but in late winter and in the melting season, the maximum concentration was at 1.5 m.

The mean water column biomass of HNF was low until mid-winter, then increased steadily throughout the remainder of the sampling period, peaking at spring overturn. This represented an 835% increase from the minimum observed in early winter to the maximum in spring (Table I). In contrast, PNF biomass peaked at autumn overturn, declined through to mid-winter, and then showed a steady increase toward spring overturn. PNF biomass increased almost six-fold from its mid-winter minimum to its spring maximum (Table I). At the depth level, HNF biomass was highest at the surface (0 m) in early winter, but the peak shifted to 1.5 m by mid-winter. In late winter and the melting season, the HNF peak occurred deeper in the water column, at 7 m. PNF exhibited a different vertical pattern: they were more abundant near the surface during early and mid-winter, but the biomass maximum also shifted to 7 m in late winter and during the melting season.

**Table I:** Seasonal and vertical variation of nanoflagellate biomass (μg C L^−1^), including HNF and PNF during the key winter periods of Lake Simoncouche in 2020–2021. Values per depth are the mean of three replicates. The water column average (W.C. avg.) is the mean (± SE) of all samples for a given date

Group	Depth (m)	Autumn overturn	Early winter	Mid-winter	Late winter	Melting season	Spring overturn
		Biomass (μg C L^−1^)					
HNF	0		43.88	39.68	44.17	71.33	
	1.5		27.46	71.10	42.49	119.77	
	3		23.19	44.84	36.83	130.7	
	4.5		20.92	27.61	51.13	152.01	
	7		18.32	31.97	126.85	233.36	
W.C. avg. ± SE		48.93 ± 5.07	26.75 ± 2.81	43.04 ± 4.60	60.29 ± 10.16	141.43 ± 18.91	217.59 ± 54.69
PNF	0		31.85	9.05	6.18	17.91	
	1.5		22.75	9.59	5.64	16.23	
	3		16.91	5.12	4.68	24.10	
	4.5		16.36	3.06	6.95	44.39	
	7		17.04	6.74	40.63	77.12	
W.C. avg. ± SE		77.10 ± 6.36	20.98 ± 1.98	6.71 ± 0.93	12.82 ± 3.82	35.95 ± 8.42	38.96 ± 21.16

**Fig. 2:**
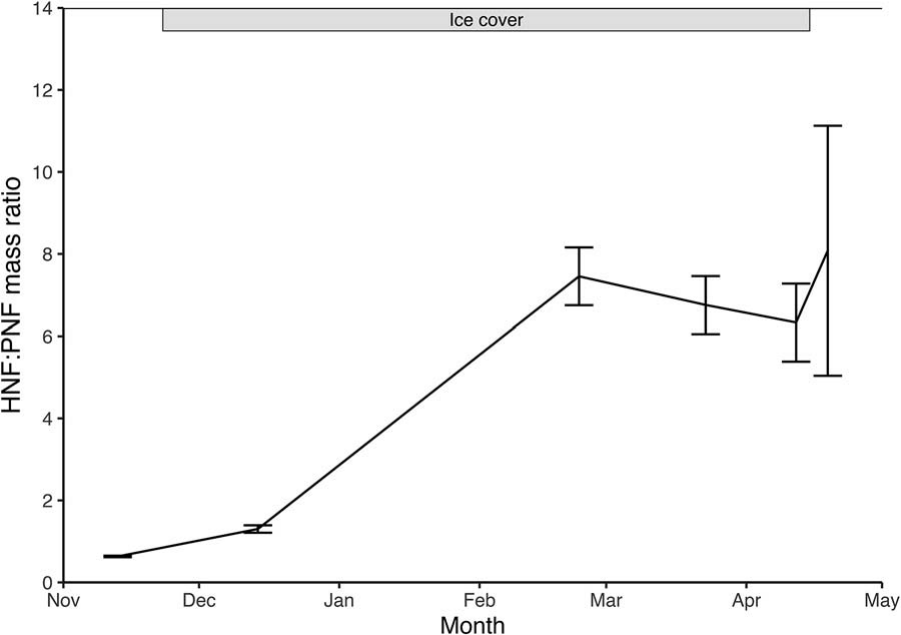
Mass ratio ± SE of mean water column biomasses of HNF and PNF, as a proxy to study the energy transfers at the base of the food web through the shoulder seasons and winter months of 2020–2021 in Lake Simoncouche.

Throughout most of the sampling period, the mean water column HNF biomass exceeded that of PNF, except during the autumn overturn. Peak HNF biomass was nearly six times higher than peak PNF biomass. As a result, the HNF:PNF biomass ratio increased over time (Fig. 2), reflecting the growing dominance of heterotrophy under the ice and into the spring overturn. This ratio was significantly lowest during the autumn overturn and rose steadily thereafter. Meanwhile, bacterioplankton biomass remained relatively stable across sampling dates and depths, whereas autotrophic picoplankton exhibited a temporal pattern similar to that of PNF, showing a carry-over of phototrophic organisms from the autumn overturn into the winter period (Supplementary Material S1: Table SI).

A total of 27 zooplankton taxa were observed across all time periods. Rotifers were the most species-rich with 18 taxa, followed by copepods with 6 species and cladocerans with 2 genera (Supplementary material S1: Table SII). Fourteen to 22 taxa were observed per sampling date (Table II), zooplankton taxa richness did not differ over time between time periods (PER-ANOVA pseudo-F_5,38_ = 2.2, Pmc = 0.07550). Six taxa dominated the community, collectively accounting for 89% of the total biomass: *Leptodiaptomus minutus* (35%), *Cyclops scutifer* (15%), *Kellicottia longispina* (12%), *Daphnia* sp. (11%), and *Bosmina* sp. (8%).

**Table II:** Taxa richness of the three main zooplankton groups at each sampling date during winter 2020–2021 in Lake Simoncouche. Values express the number of taxa

Sampling date	*Rotifera*	*Copepoda*	*Cladocera*
Autumn overturn	12	6	2
Early winter	11	5	2
Mid-winter	15	5	2
Late winter	15	4	2
Melting season	16	4	2
Spring overturn	8	4	2

**Fig. 3:**
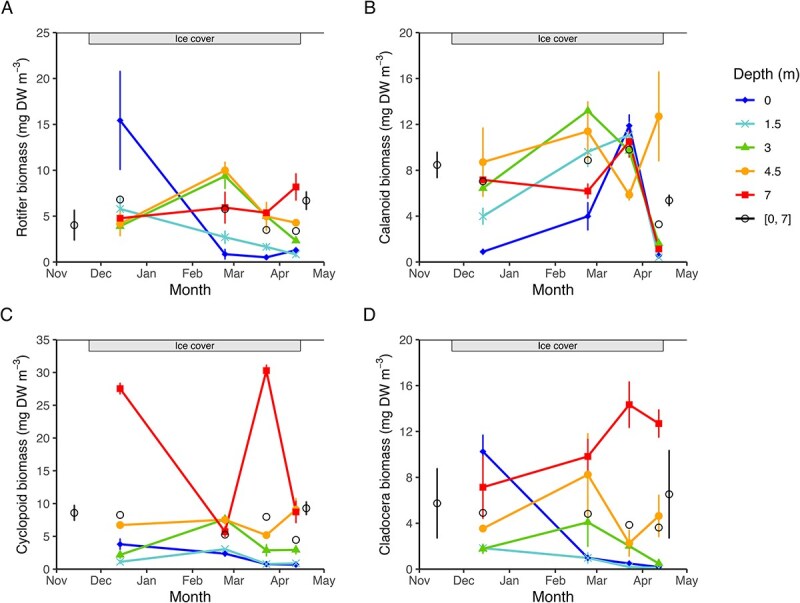
Seasonal and vertical variation in biomass (mg DW m^−3^ ± SE) of rotifers (**A**), calanoid copepods (**B**), cyclopoid copepods (**C**) and cladocerans (**D**) during the day at five fixed depths under the ice during winter 2020–2021 in Lake Simoncouche. Average water column ([0, 7] m) biomass during the autumn and spring overturns, as well as during the ice-covered period, are shown as black circles.

Mean water column biomass patterns revealed broadly similar seasonal trajectories for the four major zooplankton groups over the study period, with some distinctions (Fig. 3). Rotifer biomass increased from the autumn overturn through winter, declined slightly in late winter, and then peaked again at the spring overturn. In contrast, calanoid copepod and cyclopoid biomass remained relatively stable from the autumn overturn to the late ice-covered period, declined to a minimum in melting season, and then rose sharply by spring overturn. Cladocerans followed a similar but less pronounced trend, with biomass declining to a melting season minimum before increasing to peak levels during the spring overturn. Zooplankton biomass varied significantly with depth (Fig. 3; Supplementary Material S1: Table SIII). On average, biomass at 7 m was approximately three times higher than at 0 m and 1.5 m. Mid-depths (3 m and 4.5 m) were preferred by rotifers and calanoids, while majority of cyclopoids and cladocerans stayed at 7. An exception to the overall pattern was observed for rotifers and cladocerans in early winter, when their biomass peaked at the surface.

**Fig. 4:**
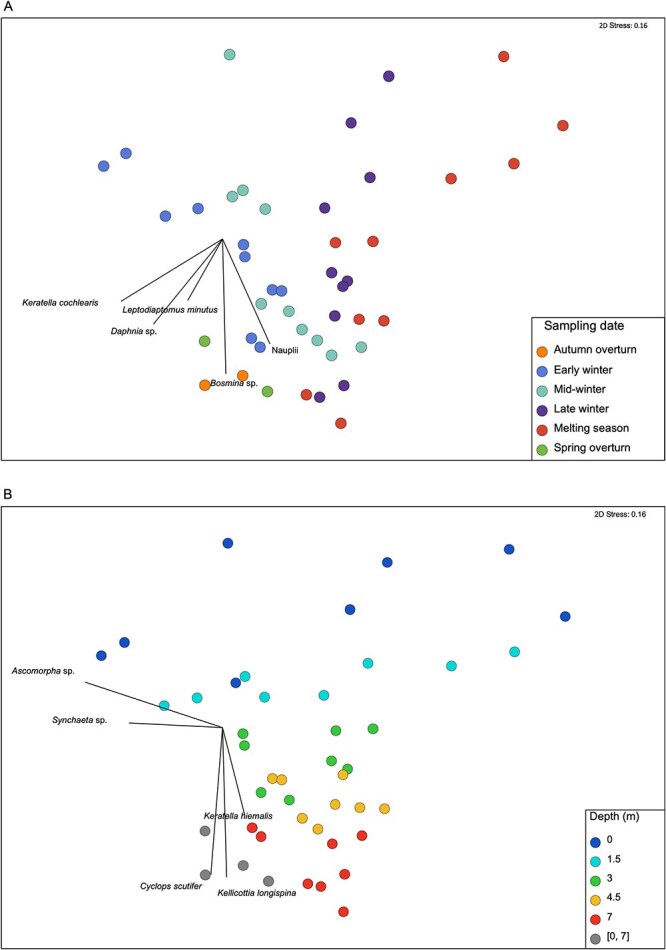
nMDS of zooplankton (cladocerans, copepods and rotifers) community structure based on Bray–Curtis dissimilarity matrix and taxon biomasses, showing differences among sampling dates (**A**) and among depths (**B**). Taxon vectors were selected based on their percent contribution to the differences between samples.

Zooplankton community structure, including rotifer, copepod and cladoceran biomasses, differed through time over the winter and with depth (PERMANOVA Pseudo-F_12,20_ = 4.5,   P< 0.0001; Supplementary Material S1: Table SIII; Fig. 4). *Keratella cochlearis* and copepod nauplii dynamics explained most of the differences in the communities between early winter and the melting season (SIMPER; Supplementary Material S1: Table SIV). *K. cochlearis* was the most abundant rotifer species accounting for 20% of total zooplankton biomass in early winter. Its biomass decreased through winter, dropping to ~ 9% of total zooplankton biomass during ice-off. Copepod nauplii were nearly absent from the water column in early winter but became very abundant, contributing ~ 34% to total community biomass in the melting season. Zooplankton community composition changed most near the lake surface, while remaining more stable in the deeper strata (Fig. 4B). Moreover, the community found in the two surface layers differed from that observed in the deepest layer, with middle layers (3 m and 4.5 m) being intermediate in their structure throughout winter (Fig. 4B). Community composition during the autumn and spring overturns was similar to the under-ice community at 7 m. The most important taxa separating the different sampling depths were *Ascomorpha* sp., *C. scutifer*, and *K. longispina* (Supplementary Material S1: Table SV). *Ascomorpha* sp. contributed ~ 9% of the biomass at the surface (directly under ice), while *C. scutifer* and *K. longispina* were most present at depth (7 m), contributing ~15% and 9%, respectively, to total biomass.

**Fig. 5:**
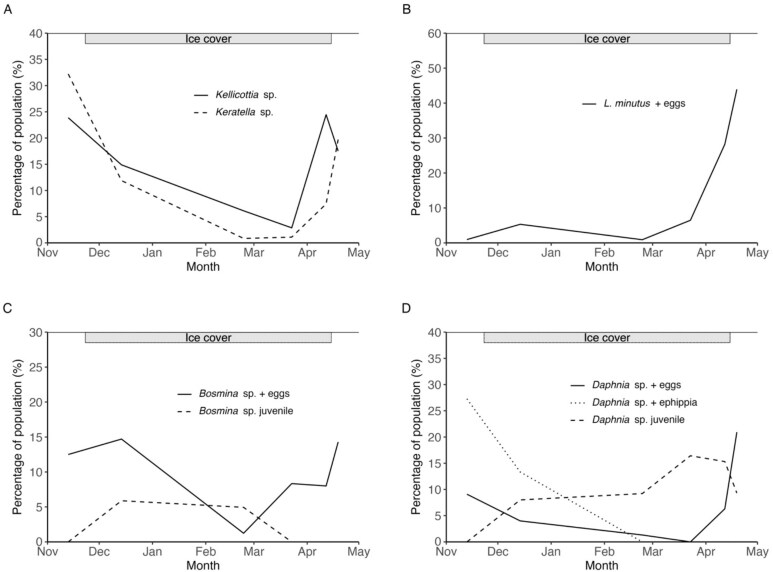
Zooplankton reproduction during winter, expressed as the percentage of rotifers (*Kellicottia sp.* and *Keratella* sp.) bearing eggs (**A**), percentage of *L. minutus* bearing eggs (**B**), percentage of *Bosmina* sp. bearing eggs or in the juvenile stage (**C**), and percentage of *Daphnia* sp. bearing ephippia, eggs or in the juvenile stage (**D**). Values express the percentage of the respective population density of each taxon.

Zooplankton egg production occurred under ice for all major groups throughout winter (Fig. 5). For rotifers *Kellicottia* sp. and *Keratella* sp., more than 20% of their populations were fecund during the melting season and the spring overturn (Fig. 5A). The observed proportion of egg-bearing females in the *L. minutus* population reached 28% in the melting season and 44% during the spring overturn (Fig. 5B). Juvenile copepods (nauplii) followed a similar pattern with highest biomass in the melting season, mostly distributed in deeper lake strata (Supplementary Material S1: Fig. S3h). Cladocerans were also fecund under ice. The proportion of *Bosmina* sp. individuals with eggs increased after ice-on in early winter. *Bosmina* sp. egg production increased to ~8% of the population in late winter and reached a maximum of 14% during the spring overturn (Fig. 5C). Juvenile *Bosmina* sp. were also observed in early and mid-winter but were absent from the water column thereafter. *Daphnia* sp. followed a similar pattern, producing eggs under ice (Fig. 5D). The proportion of fecund *Daphnia* sp. decreased during early and mid-winter, completely stopping in late winter and increasing again just before ice-off, to ~6% of the population. Egg production reached its peak during the spring overturn, with ~21% of the active *Daphnia* sp. population bearing eggs. The proportion of juvenile *Daphnia* sp. increased under ice and was highest in late winter, then decreased during the melting season and the spring overturn. Parthenogenetic resting eggs were produced during the autumn overturn and early winter, with 27% and 13% of the *Daphnia* sp. population, respectively bearing ephippia at those times (Fig. 5D).

## DISCUSSION

We observed a shift in the plankton community structure from early winter to the melting season and across depths, in line with shifts in the thermal stratification of the water column. Overall, the observed levels of zooplankton biomass and reproduction, along with shifts in microbial biomass indicate an active, responsive and dynamic under-ice biological activity rather than static, minimal or dormant system. Our results agree with previous winter plankton studies that have shown active and abundant zooplankton communities under the ice in boreal lakes (Salonen *et al.,* 2009; Hampton *et al.,* 2017; Grosbois *et al.,* 2020; Hébert *et al.,* 2021). Very few studies have, however, studied the changes in vertical distribution of zooplankton under ice over a period of several months, and our results provide new high-resolution, two-dimensional (time and depth) information on winter ecology of plankton in boreal lakes.

Heterotrophy became the main energy pathway during winter, making microplankton and the microbial loop essential components of the active food web. The classical summer food web, fueled by solar radiation through primary producers, was hindered by ice and snow that block light (Prowse and Stephenson, 1986; Pernica *et al*., 2017), and led to a decrease in the biomass of photo-autotrophic organisms, including picoautotrophs and PNF as predicted by our first hypothesis (H1). Larger phytoplankton were not examined in this study, but under-ice chlorophyll-*a* concentrations were comparable to those measured in a previous year when phytoplankton cell counts averaged ~200 000 cells/mL (Imbeau *et al*., 2021). This is two orders of magnitude lower than the mean abundance of PNF observed in our study, suggesting that PNF were the primary source of autotrophic resources for zooplankton. In the reduction of solar radiation and primary production, the under-ice food web was however rather enabled by the recycling and uptake of organic matter and dissolved carbon compounds by heterotrophic micro-organisms (Sommer *et al.,* 2012; Kivilä *et al.,* 2023). The mass ratio of HNF:PNF shifted from nearly equal biomass of pigmented to non-pigmented flagellates in autumn to a dominance of obligate heterotrophs with the progression of winter, especially at deeper depths. This shift indicated the elimination of photosynthetic pigments in the reduction of light and follows the seasonal pattern and HNF:PNF proportions seen in other ice-covered lakes (Rautio *et al.,* 2011; Salmi and Salonen, 2016; Hébert *et al.,* 2021). By downregulating pigment production and its associated energetic costs (Blankenship, 2002), nanoflagellates appeared to shift toward a heterotrophic nutritional strategy, deriving energy from the consumption of bacteria or organic matter, which can be seen as an adaptation to the limited solar energy characteristic of winter lake ecosystems. These heterotrophs likely replaced autotrophic plankton in the food web and served as a resource for the abundant rotifers, copepods and cladocerans, although crustacean zooplankton likely also relied on storage lipids accumulated during autumn as an additional energy source (Mariash *et al*., 2016; Schneider *et al.,* 2017; Grosbois *et al.,* 2017).

Despite high zooplankton species number under the ice, the values were lower than in summer (Grosbois *et al.,* 2020) supporting our second hypothesis (H2). *Mecocyclops edax*, *Diaphosoma* sp., *Holopedium* sp. and *Leptodora kindtii* that contribute > 15% of the summer crustacean production in Lake Simoncouche, were entirely absent from the water column, including the autumn and spring shoulder seasons when there was no ice. While our data and existing winter limnology studies indicate that lakes can support high zooplankton richness in winter (reviewed by Hampton *et al*., 2017), some species clearly struggle under conditions of low or absent primary production and persistently low temperatures. For example, the absence of *L. kindtii* in winter, despite the potential availability of prey, underscores its affinity for temperatures exceeding 10°C (Wojtal-Frankiewicz *et al*., 2015). If a taxon was encountered during the autumn overturn (21 days before ice-in, water temperature 5.8°C), it usually persisted throughout the winter. Only the cyclopoid *Epischura lacustris*, which was abundant under the ice in early winter, disappeared from the water column by mid-winter. However, some taxa appeared during winter, including the rotifers *Conochilus unicornis*, *Filinia longiseta*, *F. terminalis*, and *Hexarthra mira* in mid-winter, and *Keratella serrulata* during the melting season. Most of these had low abundance and were possibly missed on other sampling dates.

Similar to the stable zooplankton species richness observed throughout the winter, the relative abundance and biomass of major taxonomic groups also remained consistent from autumn overturn through to the melting season. This contrasts with the seasonal dynamics predicted by the PEG model (Sommer *et al.,* 2012). Although high-frequency winter sampling is rare, the few studies that have focused on winter seasonality report similar patterns (Rigler *et al*., 1974; Rautio *et al.,* 2011). Despite the persistent scarcity of algal resources, depletion of lipid reserves, and declining oxygen concentrations (Jansen *et al*., 2021), the biomass of rotifers, copepods and cladocerans remained stable. However, individual species within these groups exhibited more variability, contributing to the temporal shifts in community structure observed in our study. Environmental variables, such as temperature, oxygen saturation and chlorophyll-*a* concentration, were all low under the ice, until the ice began to melt, likely representing demanding ecological conditions for zooplankton. Conditions became more favorable at the ice melt, when picoautotrophs, PNF and chlorophyll-*a* concentration increased in the water column due to increased light penetration through the ice. However, this improvement did not coincide with a noticeable rise in overall zooplankton abundance. Our spring overturn sampling may have occurred too soon after ice-off to detect a biomass response. However, as shown earlier for Lake Simoncouche, the biomass of *L. minutus,* the lake’s dominant crustacean zooplankton, actually declined after ice-out (Schneider *et al.,* 2017).

Based on biomass, the most successful species in the winter-active zooplankton community were *L. minutus* (35% of total biomass), *C. scutifer* (15%) and *Daphnia* sp. (11%). These species can remain active under ice by using lipid reserves accumulated during the autumn and individuals are brightly colored by carotenoids from algal derived diet, that are used as antioxidant to protect the fat reserves (Grosbois *et al.,* 2017; Schneider *et al.,* 2017). Accumulated lipids enable overwintering and under-ice reproduction while reducing the reliance on active feeding. However, some taxa that do not typically accumulate lipids, such as *K. longispina* (12% of total biomass) and *Bosmina* sp. (8%), also ranked high in biomass. These species likely relied on alternative food sources, such as nanoflagellates. HNF may strongly improve the food uptake of predominantly herbivorous consumers at times when the phytoplankton is deficient (Klein Breteler *et al*., 1999).

Vertical patterns of zooplankton biomass revealed substantial differences among depths, which persisted throughout the entire ice-covered period. Surface waters, characterized by icy temperatures, were generally avoided by zooplankton, except for a few species in early winter, when the ice was still thin and PAR levels were sufficient to support photosynthesis (Hoppe *et al*., 2024). This likely explains the high chlorophyll-*a* concentrations observed in the upper layers in early winter, which seemed to have attracted some zooplankton. Apart from the early winter, surface waters did not provide a favorable habitat due to the low phototrophic biomass, supporting predictions from H3 for mid-winter. We predicted that during the melting period when light increased, increased phytoplankton production should support zooplankton biomass at the surface. However, chlorophyll-*a* concentration remained low at the surface during that time. A rapid transition from low to high light levels at snow melt likely created stressful conditions immediately beneath the ice, potentially causing photoinhibition and damaging the photosynthetic machinery of phototrophs, consistent with findings from a companion study (Kivilä *et al.,* 2023). Zooplankton may have also avoided the surface to escape visual predation. While direct evidence for such avoidance behavior is largely limited to the summer months (Sastri *et al.,* 2014), it is possible that the low light levels potentially present at 0 m—but not anymore recorded by our PAR sensor at 1.5 m during most of the winter—were sufficient to enable visual predation, prompting zooplankton to descend deeper in the water column.

Zooplankton layering at the deeper depths was likely driven by the combined effects of diet, temperature and oxygen. Under the ice, chlorophyll-*a* concentration peaked at 1.5 m and was next highest at 3-m depth. The higher availability of phototrophic diet may have attracted and explained the preference of rotifers to these depths. High oxygen concentration was another reason to favor the mid water column. In mid-winter, the bottom water was hypoxic (2.5%, 0.34 mg L^−1^) and majority of copepods were found at mid water column depths with more oxygen. None of the species, however, were absent from the bottom 7 m depth suggesting tolerance to low oxygen. Cladocerans seemed exceptionally unbothered by this hypoxia, maintaining a high abundance at the 7 m depth. This taxonomic group is sensitive to low temperatures but can withstand low oxygen concentrations and follows the availability of food resources (Vanderploeg *et al.,* 2009; Lampert *et al.,* 2010). The higher temperatures deeper in the water column (4.5 and 7 m) was likely a reason for cyclopoids and cladocerans to be most abundant at these depths. Temperature is a key driver of invertebrate metabolism, activity, growth, and reproduction, all of which generally increase with rising temperatures (*e.g.*  Brown *et al*., 2004). Even during the melting season, when chlorophyll-*a* concentrations substantially increased at 1.5 and 3 m depths, these zooplankton did not migrate upward to the resource-rich layers. This suggest that the higher temperatures and abundant nanoflagellate community (and likely also ciliates, not studied here) at deeper depths provided the resources required by zooplankton. Our sampling was conducted during daylight hours, so we cannot exclude the possibility that some zooplankton engaged in diel vertical migration (DVM). However, no light was detected by the sensor (1.5 m depth) during the period from early December to late March, which includes three of the sampling dates. Previous studies reporting under-ice DVM were typically conducted later in spring or under ice and snow conditions that allowed sufficient light penetration to stimulate zooplankton’s vertical movement (Cunningham, 1972; Chiapella *et al*., 2021). It is also possible that DVM was triggered by stimuli other than PAR, such as endogenous rhythms of zooplankton, however, confirming this would require further studies.

Another likely reason for the preference for bottom depths was the lateral inflow of water from the littoral zone, which transported oxygen-rich water and additional resources to deeper layers. Rising oxygen concentrations at 7 m, along with data from five sensor chains positioned between the sampling site and the littoral zone (unpublished data), confirmed the development of this flow in late winter. The inflow increased oxygen saturation to ~45% at 7 m, coinciding with a rise in zooplankton biomass at that depth. The current also transferred organic matter and nutrients from the littoral to the deeper pelagic zone (Kivilä *et al.,* 2023), supplying heterotrophic organisms with fresh resources, including littoral benthic algae consumed by overwintering copepods (Karlsson and Säwström, 2009). PNF were likely transported by this flow as well, as indicated by their elevated biomass near the bottom during late winter, three weeks before ice-out, when PAR was still absent from deeper depths. These inputs likely supported microbial and metazoan food webs. While such under-ice currents have been documented previously (Cortés and MacIntyre, 2020; MacIntyre *et al*., 2018), their ecological consequences for plankton communities remain largely unexplored (Jansen *et al.,* 2021).

Zooplankton from all three major taxonomic groups reproduced under the ice, as indicated by female fecundity and the presence of juvenile stages. These findings align with our final two hypotheses (H4 and H5) and with earlier studies showing that under-ice zooplankton production typically peaks in early and late winter (Rautio *et al.,* 2011; Grosbois *et al.,* 2020). For example, cladoceran reproduction in early winter can account for up to half of their annual production (Grosbois *et al.,* 2020), and for some copepods, the main reproductive event occurs in spring beneath the ice (Rigler *et al*., 1974; Schneider *et al.,* 2017). Our results are consistent with these patterns and further demonstrate that rotifers also reproduce during winter. Rotifer fecundity was high in both early winter and the melting season, with approximately one-quarter of the *Kellicottia* population bearing eggs. This number likely underestimates actual reproduction, as some eggs may have detached and been lost during sample preservation. Copepod nauplii abundance also peaked during this transitional open-water period, showing a strong preference for deeper layers. During their early life stages, these nauplii rely on maternal lipid reserves, which are rich in essential fatty acids (Schneider *et al.,* 2017). As a result, the nauplii did not need to migrate toward surface layers where primary production occurred (Kivilä *et al.,* 2023) but could remain in the darker bottom waters, where they were protected from visual predators. Overall, zooplankton reproductive activity occurred immediately before (spring) or during periods of elevated chlorophyll-*a* concentrations (autumn and spring), highlighting the critical role of algal diets and their associated essential fatty acids in supporting successful reproduction (Müller-Navarra *et al*., 2000).

## CONCLUSION

We studied the microbial loop and zooplankton community dynamics in ice-covered boreal Lake Simoncouche during winter, focusing on three key objectives. We inferred energy transfer at the base of the food web using PNF and HNF, and observed decreases in photo-autotrophy with progressing winter and with depth. Moreover, the increasing HNF:PNF ratio throughout winter peaked before ice melt, indicating heightened metabolic activity within the water column, with heterotrophy as the dominant energy pathway. Our results confirmed significant temporal and vertical heterogeneity in winter active zooplankton communities. Despite an overall biomass decline in all three zooplankton groups over winter (rotifers, copepods, and cladocerans), they were all represented throughout winter 2020–2021. While cladoceran biomass decreased under ice, they remained present in the water column, using deeper strata as thermal refuges and providing evidence for active overwintering of species such as *Daphnia* sp. and *Bosmina* sp. Zooplankton species previously identified in the lake during summer were absent in winter, hinting at the challenging conditions for heterotrophic metabolism in the ice-covered water column with cold temperatures, low light, oxygen levels and food availability. Finally, our study of zooplankton fecundity and reproduction highlights the ecological significance of winter as part of the annual successional cycle. In line with what is known about the reproductive patterns of *L. minutus*, we observed an increase in copepod nauplii abundance toward the end of winter, providing evidence of under-ice reproduction by copepods. Moreover, we reported egg production during the ice-covered season for four rotifer species. Our assessment of plankton community dynamics in an ice-covered boreal lake addresses the urgent need for baseline winter data in plankton research. This research is one of several recent papers which lay the groundwork for future studies that will address the long-term implications of climate change on ice-covered aquatic ecosystems.

## Supplementary Material

Supplementary_material_fbaf035

## Data Availability

The data that support the findings of this study are openly available at Borealis, the Canadian Dataverse Repository: doi: 10.5683/SP3/MOM5UD

## Notes

Corresponding editor: Lars-Anders Hansson
