# Supplementary material for: Winter plankton dynamics in a boreal lake: community structure, vertical distribution and reproduction under ice
Source: J Plankton Res. 2025 Aug 17;47(5):fbaf035. doi: 10.1093/plankt/fbaf035 (PMC12358046; doi:10.1093/plankt/fbaf035)
Supplement: Supplementary_material_fbaf035 [file supplementary_material_fbaf035.docx]

**SUPPLEMENTARY MATERIAL 1**

**Additional tables and figures**

Table SI: Seasonal and vertical variation of microbial biomass (μg C L^-1^), including bacterioplankton and pigmented picoplankton, during the key winter periods of Lake Simoncouche in 2020-2021. Values by depth represent the mean of three replicates. The water column average is the mean (± SE) of all replicates across all depths for a given date.

| **Group** | **Depth (m)** | **Autumn overturn** | **Early winter** | **Mid-winter** | **Late winter** | **Melting ice** | **Spring overturn** |
| --- | --- | --- | --- | --- | --- | --- | --- |
|  |  | **Biomass (μg C L^-1^)** | | | | | |
| **Bacterioplankton** | 0 | - | 13.02 | 13.84 | 7.71 | 6.79 | - |
|  | 1.5 | - | 12.54 | 10.19 | 7.34 | 8.61 | - |
|  | 3 | - | 12.58 | 10.67 | 7.25 | 9.04 | - |
|  | 4.5 | - | 13.66 | 12.12 | 7.34 | 11.62 | - |
|  | 7 | - | 14.05 | 8.73 | 9.98 | 14.35 | - |
| W.C. Avg. ± SE |  | 26.58 ± 1.67 | 13.17 ± 0.26 | 11.11 ± 0.68 | 7.92 ± 0.37 | 10.08 ± 0.86 | 9.16 ± 0.36 |
|  |  |  |  |  |  |  |  |
| **Picoplankton** | 0 | - | 2.84 | 4.91 | 2.36 | 1.46 | - |
|  | 1.5 | - | 5.25 | 3.04 | 4.2 | 3.87 | - |
|  | 3 | - | 5.29 | 4.36 | 3.5 | 5.43 | - |
|  | 4.5 | - | 3.59 | 10.53 | 4.77 | 9.59 | - |
|  | 7 | - | 1.8 | 3.95 | 7.18 | 20.88 | - |
| W.C. Avg. ± SE |  | 6.99 ± 0.55 | 3.91 ± 0.40 | 5.24 ± 0.67 | 3.99 ± 0.37 | 7.25 ± 1.41 | 2.93 ± 3.87 |

**SUPPLEMENTARY MATERIAL 1 (continued)**

Table SII: Zooplankton taxa encountered under the ice and during the winter shoulder seasons 2020-2021 in Lake Simoncouche.

| **Rotifera** | **Copepoda** | **Cladocera** |
| --- | --- | --- |
| *Hexarthra mira* | *Epischura lacustris* | *Bosmina* sp. |
| *Pompholyx sulcata* | *Leptodiaptomus minutus* | *Daphnia* sp. |
| *Testudinella* sp. | *Tropocyclops prasinus* |  |
| *Conochilloides* sp. | *Cyclops scutifer* |  |
| *Conochilus unicornis* | *Eucyclops speratus* |  |
| *Filinia terminalis* | *Mesocyclops edax* |  |
| *Filinia longiseta* |  |  |
| *Gastropus stylifer* |  |  |
| *Polyarthra* sp. |  |  |
| *Ploesoma* sp*.* |  |  |
| *Synchaeta* sp. |  |  |
| *Ascomorpha* sp. |  |  |
| *Lepadella* sp. |  |  |
| *Kellicottia longispina* |  |  |
| *Kellicottia bostoniensis* |  |  |
| *Notholca squamula* |  |  |
| *Keratella serrulata* |  |  |
| *Keratella hiemalis* |  |  |
| *Keratella cochlearis* |  |  |

**SUPPLEMENTARY MATERIAL 1 (continued)**

Table SIII: Per-ANOVA results for total zooplankton biomass, cladocerans biomass, calanoid and cyclopoid copepod biomass, rotifer biomass, and HNF:PNF mass ratio.

| **Group** | **Source** | **df** | **SS** | **MS** | **Pseudo-F** | **perms** | **P(perm)** |
| --- | --- | --- | --- | --- | --- | --- | --- |
| HNF:PNF | Date | 5 | 25529 | 5105.9 | 41.46 | 9933 | 0.0001 |
|  | Residuals | 60 | 7390.1 | 123.2 |  |  |  |
|  | Total | 65 | 32919 |  |  |  |  |
| Rotifer biomass | Date | 3 | 11816 | 3938.5 | 14.98 | 9909 | 0.0001 |
|  | Depth | 4 | 13666 | 3416.4 | 13 | 9912 | 0.0001 |
|  | Date*Depth | 12 | 9741.1 | 811.8 | 3.09 | 9859 | 0.0001 |
|  | Residuals | 20 | 5256.9 | 262.8 |  |  |  |
|  | Total | 39 | 40479 |  |  |  |  |
| Calanoid biomass | Date | 3 | 15977 | 5325.6 | 81.96 | 9951 | 0.0001 |
|  | Depth | 4 | 6759.4 | 1689.9 | 26.01 | 9931 | 0.0001 |
|  | Date*Depth | 12 | 10646 | 887.2 | 13.65 | 9903 | 0.0001 |
|  | Residuals | 20 | 1299.5 | 65 |  |  |  |
|  | Total | 39 | 34682 |  |  |  |  |
| Cyclopoid biomass | Date | 3 | 3296.1 | 1098.7 | 5.32 | 9952 | 0.0004 |
|  | Depth | 4 | 10303 | 2575.9 | 12.47 | 9944 | 0.0001 |
|  | Date*Depth | 12 | 11880 | 990 | 4.79 | 9929 | 0.0001 |
|  | Residuals | 20 | 4132 | 206.6 |  |  |  |
|  | Total | 39 | 29612 |  |  |  |  |
| Cladoceran biomass | Date | 3 | 5030.8 | 1676.9 | 6.87 | 9924 | 0.0002 |
|  | Depth | 4 | 14187 | 3546.7 | 14.54 | 9933 | 0.0001 |
|  | Date*Depth | 12 | 28378 | 2364.9 | 9.7 | 9924 | 0.0001 |
|  | Residuals | 17 | 4146.7 | 243.9 |  |  |  |
|  | Total | 36 | 52153 |  |  |  |  |
| Zooplankton community | Date | 3 | 10271 | 3423.6 | 18.203 | 9917 | 0.0001 |
|  | Depth | 4 | 12841 | 3210.3 | 17.069 | 9906 | 0.0001 |
|  | Date*Depth | 12 | 10163 | 846.91 | 4.5031 | 9856 | 0.0001 |
|  | Residuals | 20 | 3761.5 | 188.07 |  |  |  |
|  | Total | 39 | 37036 |  |  |  |  |

**SUPPLEMENTARY MATERIAL 1 (continued)**

Table SIV: Summary of SIMPER (percentage of similarity) for zooplankton community structure between early winter (EW) and the melting season (MS) for taxa that cumulatively contributed up to 70% dissimilarity between dates. Av. abun.: average abundance; Av. diss.: average dissimilarity; Diss/SD: dissimilarity divided by standard deviation; Contrib.%: percentage of contribution; Cum.%: cumulated percentage of contribution.

| Species | Av. abun. EW | Av. abun. MS | Av. diss | Diss/SD | Contrib. (%) | Cum. |
| --- | --- | --- | --- | --- | --- | --- |
|  |  |  |  |  |  | (%) |
| *Keratella cochlearis* | 3.54 | 1.24 | 6.02 | 1.78 | 12.4 | 12.4 |
| Nauplii spp. | 0.44 | 2.25 | 4.17 | 1.82 | 8.59 | 21 |
| *Ascomorpha* sp. | 2.14 | 0.64 | 4.07 | 1.17 | 8.38 | 29.38 |
| *Polyarthra* sp. | 1.83 | 0.59 | 3.62 | 1.32 | 7.46 | 36.83 |
| *Leptodiaptomus minutus* | 1.96 | 1.11 | 3.11 | 1.54 | 6.4 | 43.23 |
| *Daphnia* sp. | 1.57 | 1.03 | 3.06 | 1.4 | 6.31 | 49.54 |
| *Synchaeta* sp. | 1.75 | 0.67 | 3.04 | 1.26 | 6.27 | 55.81 |
| *Cyclops scutifer* | 2.26 | 1.7 | 3.01 | 1.18 | 6.2 | 62.01 |
| *Kellicottia longispina* | 1.73 | 1.71 | 2.71 | 1.33 | 5.58 | 67.59 |
| *Tropocyclops prasinus* | 1.67 | 1.56 | 2.52 | 1.56 | 5.19 | 72.78 |

**SUPPLEMENTARY MATERIAL 1 (continued)**

Table SV: Summary of SIMPER (percentage of similarity) for zooplankton community structure between 1.5 m and 7 m for taxa that cumulatively contributed up to 70% dissimilarity between dates. Av. abun.: average abundance; Av. diss.: average dissimilarity; Diss/SD: dissimilarity divided by standard deviation; Contrib.%: percentage of contribution; Cum.%: cumulated percentage of contribution.

| Species | Av. abun. 1.5 m | | Av. abun. 7 m | Av. diss | Diss/SD | Contrib. (%) | Cum. |
| --- | --- | --- | --- | --- | --- | --- | --- |
|  |  |  |  |  |  |  | (%) |
| *Cyclops scutifer* | | 1.1 | 3.91 | 6.18 | 4.09 | 13.15 | 13.15 |
| *Bosmina* sp. | | 0.07 | 2.06 | 4.37 | 2.48 | 9.29 | 22.44 |
| *Daphnia* sp. | | 0.58 | 2.21 | 4.05 | 1.54 | 8.61 | 31.05 |
| Nauplii spp. | | 0.37 | 1.89 | 3.43 | 2.24 | 7.29 | 38.35 |
| *Kellicottia longispina* | | 1.24 | 2.61 | 3.13 | 1.72 | 6.65 | 45 |
| *Kellicottia bostoniensis* | | 0 | 1.39 | 2.95 | 1.54 | 6.28 | 51.29 |
| *Tropocyclops prasinus* | | 0.84 | 1.89 | 2.73 | 1.75 | 5.8 | 57.09 |
| *Ascomorpha* sp. | | 1.46 | 0.61 | 2.69 | 2.38 | 5.71 | 62.8 |
| *Keratella hiemalis* | | 1.95 | 3.13 | 2.65 | 1.72 | 5.63 | 68.43 |
| *Filinia terminalis* | | 0.11 | 1.14 | 2.41 | 1.33 | 5.12 | 73.55 |

**SUPPLEMENTARY MATERIAL 1 (continued)**


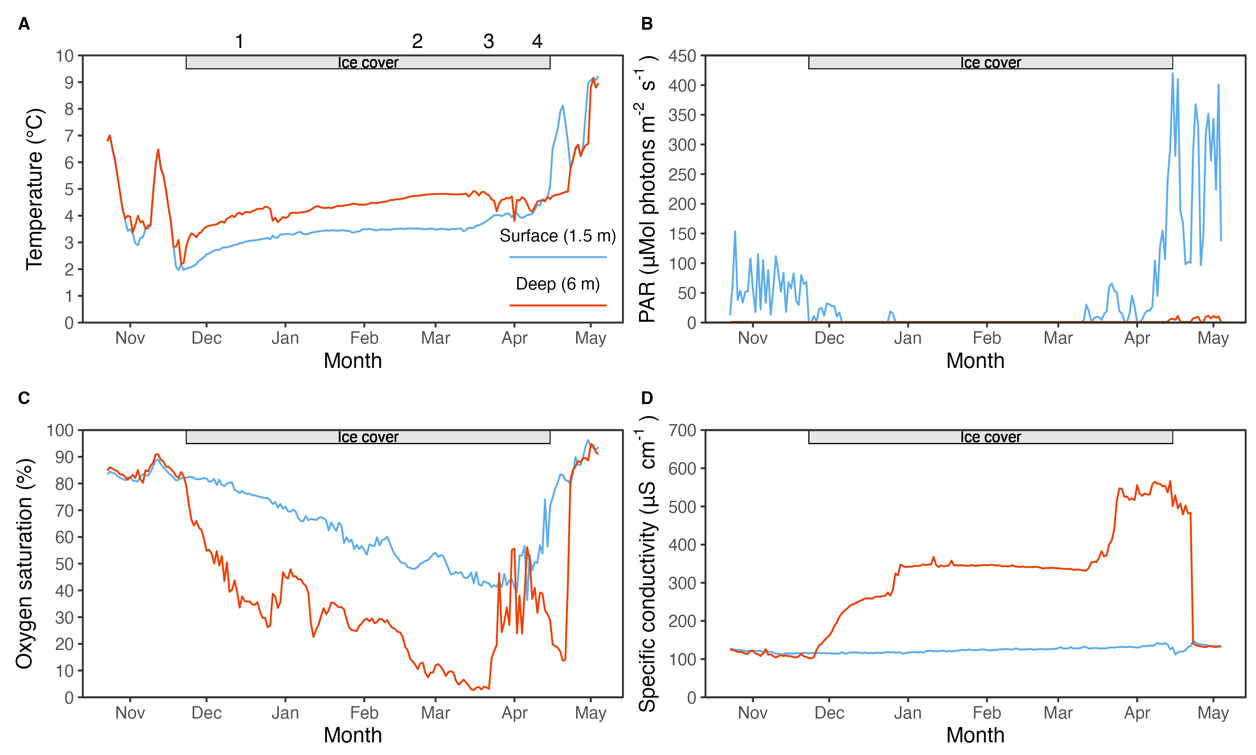


Figure S1: Daily mid-day (12:00 pm) temperatures (A), photosynthetically active radiations (B), oxygen saturation (C) and specific conductivity (D) collected from two long term RBR Concerto multisensors moored near the surface (1.5 m calculated from the top of the ice) and the bottom (6 m) of the water column in Lake Simoncouche during winter 2020-2021. Key winter periods considered in this study are indicated with numbers in panel A: early winter (1), mid-winter (2), later winter (3) and melting season (4).

**SUPPLEMENTARY MATERIAL 1 (continued)**


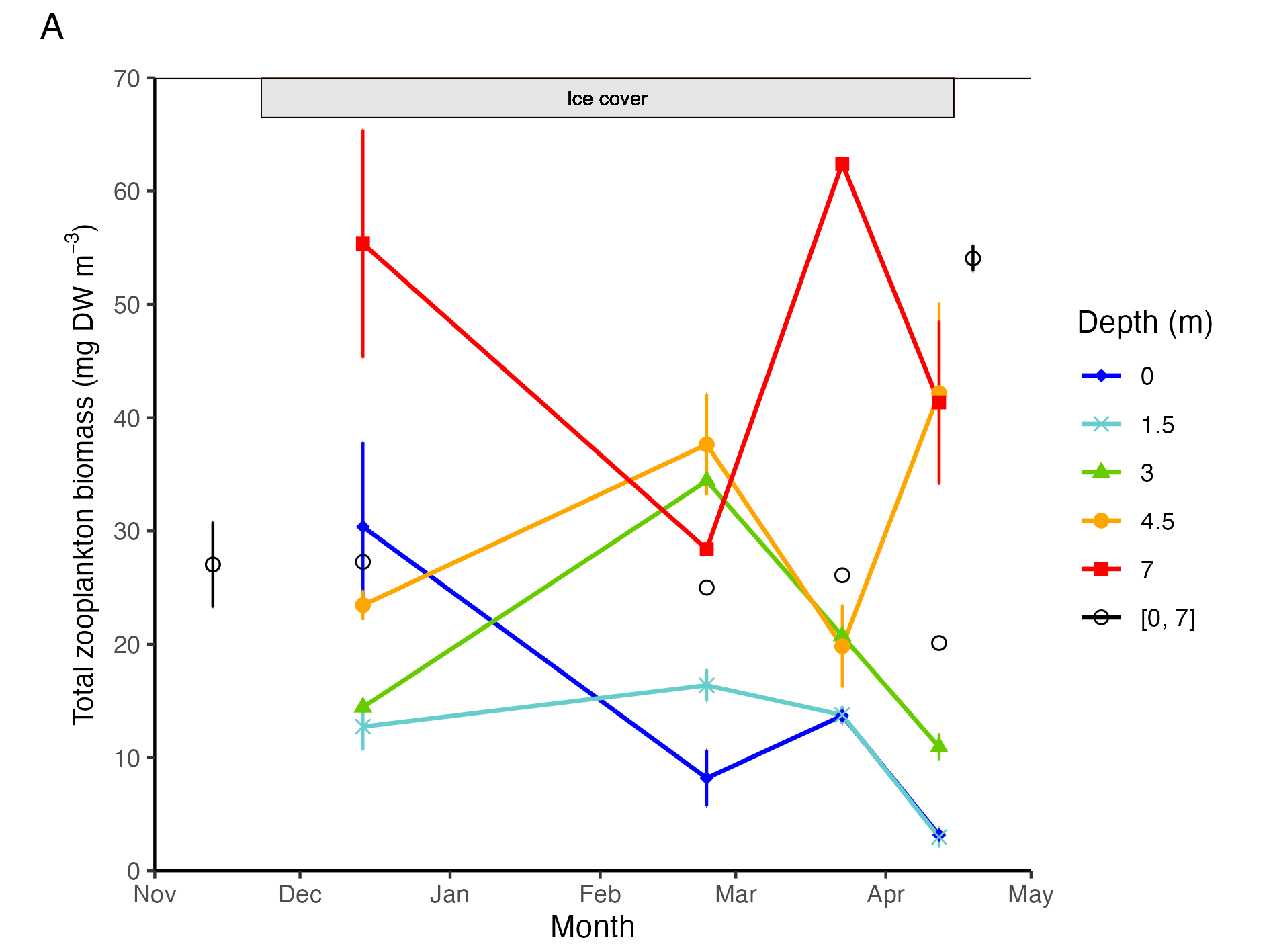


Figure S2: Zooplankton biomass (mg DW m^-3^ ± SE) during the day at five fixed depths under the ice in winter 2020-2021 in Lake Simoncouche. Biomass during the autumn and spring overturn is shown as an average for the water column ([0, 7] m). All taxa of cladocerans, copepods and rotifers are pooled together, and the black circles represent the average water column biomass, as a mean of all samples for a given date.

**SUPPLEMENTARY MATERIAL 1 (continued)**


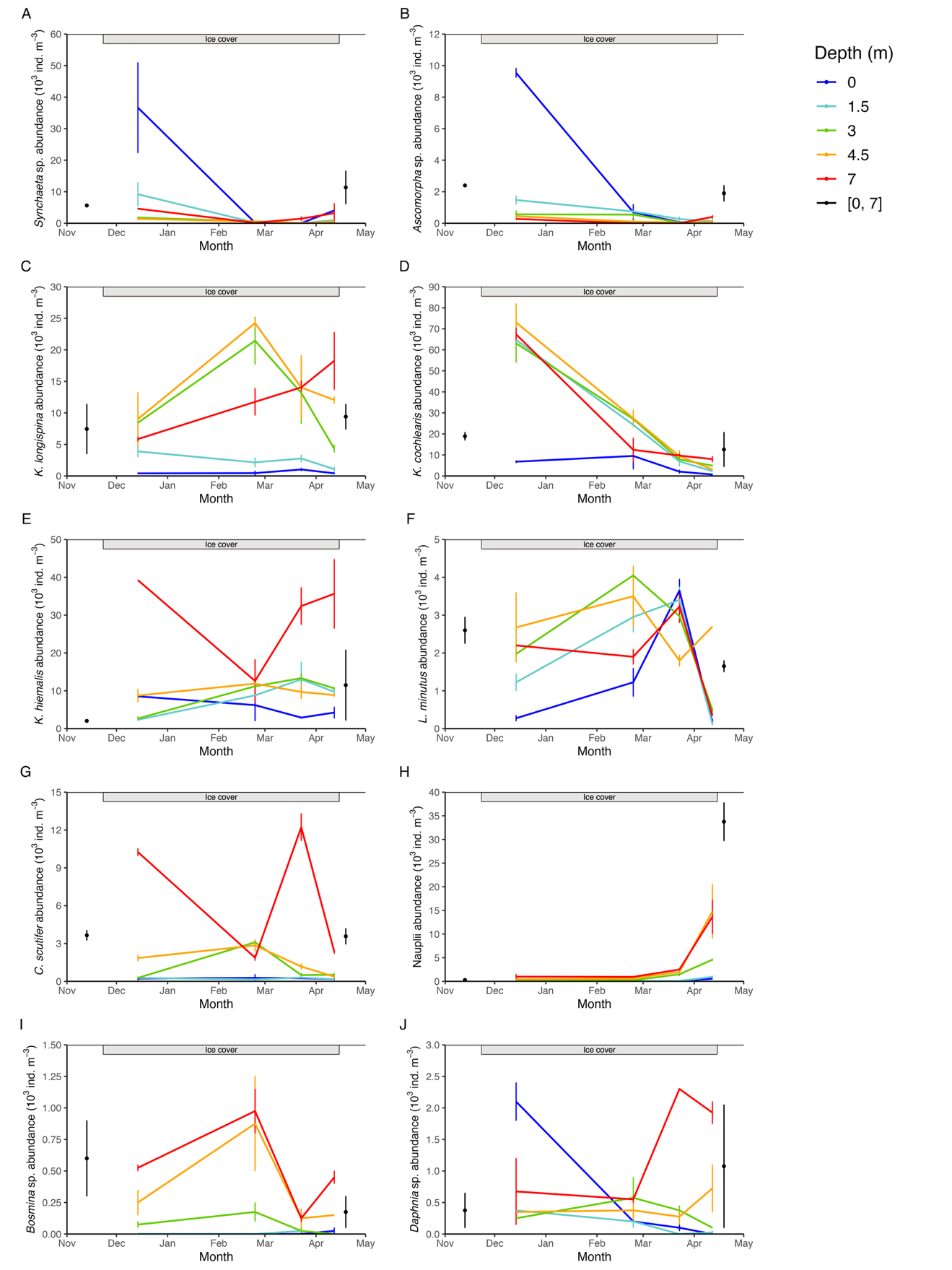


Figure S3: Taxa-specific abundance (10^3^ ind. m^-3^ ± SE) of *Synchaeta* sp. (A), *Ascomorpha* sp. (B), *K. longispina* (C), *K. cochlearis* (D), *K. hiemalis* (E), *L. minutus* (F), *C. scutifer* (G), copepod nauplii (H), *Bosmina* sp. (I), and *Daphnia* sp. (J) during the day at five fixed depths under the ice in winter 2020-2021 in Lake Simoncouche. Abundance during the autumn and spring overturn is shown as an average for the water column ([0, 7] m).

**SUPPLEMENTARY MATERIAL 2**

**Bacterioplankton, nanoflagellate and picoplankton sample processing**

Sample for analyses of the microbial components were prepared immediately after arriving at the laboratory; a 10 mL aliquot of water from each sample bottle was preserved with 2 mL of 0.2 µm filtered formaldehyde solution (2% final concentration) for bacteria enumeration and subsequent biomass analyses. Another aliquot of 50 mL was preserved with 5 mL filtered glutaraldehyde solution (1% final concentration) for a combined sample of nanoflagellates and picoplankton. Preserved samples were stored at 4°C and slides were prepared within 48h. Slides for bacteria were prepared from 1 mL of lake water that was first stained with nucleic acid–staining fluorochrome 4',6-diamidino-2-phenylindole (DAPI) and then filtered through a 0.2 µm Nucleopore membrane (Whatman, UK). Filters were permanently mounted on microscope slides with immersion oil, as in Rautio *et al.* (2011). Combined nanoflagellates and picoplankton slides were prepared in a similar fashion, except that 25 mL of lake water was filtered through a 0.6 µm black polycarbonate membrane (Whatman, UK). Samples were stored at -20°C until microscopic examination.

Enumeration of the microbial organisms was done at 100X magnification using an Axio Observer A1 (Zeiss, Germany) microscope with ultraviolet (UV) illumination. DAPI strongly binds to AT-rich regions in DNA and is fluorescent under UV light. DAPI-stained cells therefore appear bright in UV illuminated microscopy, allowing cell enumeration and abundance estimates. Bacterioplankton were enumerated until at least 400 individuals per slide and the total number of fields required to count the bacteria was noted. Bacterial carbon biomass was calculated using an average measured cell volume of 0.1 µm^3^ and a volume–biomass conversion factor of 0.308 pg C µm^3^ (Fry, 1988).

A combination of UV excitation and light filters was used to visually distinguish between pigmented (PNF) and heterotrophic (HNF) nanoflagellates. The fluorescence from DAPI is detected in both PNF and HNF when excited with UV light, and all the stained cells are clearly defined and emit bright light. A green light filter blocks DAPI fluorescence and thus, HNF are no longer clearly visible. The photosynthetic pigments produced by the PNF have fluorescent properties when exposed to UV light, with emission wavelengths that allow the bright image of the pigmented cells to appear through the green filter. This method of combined UV illumination and green light filters allowed us to visually assess whether single cell organisms are producing pigments or not and in the context of this study, whether nanoflagellates were heterotrophic (HNF) or pigmented and thus photo-autotrophic (PNF). Nanoflagellates were enumerated in three different size categories (diameter 2–5, 5–10, and > 10 µm) to

**SUPPLEMENTARY MATERIAL 2 (continued)**

allow volume estimation and subsequent carbon content for biomass conversion, using a conversion factor of 0.22 pg C µm^3^ (Børsheim *et al.*, 1990).

Photo-autotrophic picoplankton was counted in conjunction with the nanoflagellates (NF) using the UV light and green filter method. Their robust cell wall prevents the DAPI from reaching the DNA, but their photosynthetic pigments are fluorescent under UV light, and their smaller size (< 2 μm) differentiates them from the NF. Picoautotroph carbon biomass was calculated using an average cell volume of 4.2 µm^3^ and the same carbon conversion factor as for nanoflagellates.

**References**

Børsheim, K. Y., Bratbak, G. and Heldal, M. (1990) Enumeration and biomass estimation of planktonic bacteria and viruses by transmission electron microscopy. *Applied and Environmental Microbiology,* **56,** 352-356.

Fry, J. C. (1988) Determination of biomass. In: B. Austin (ed) *Methods in aquatic bacteriology.* Wiley, Chinchester, NY, pp. 27-72.

Rautio, M., Mariash, H. and Forsström, L. (2011) Seasonal shifts between autochthonous and allochthonous carbon contributions to zooplankton diets in a subarctic lake. *Journal of Limnology,* **56,** 1513-1524.
